# A Suggestion for the Weather Bureau

**Published:** 1912-01

**Authors:** 


### A Suggestion for the Weather Bureau

RESIDENTS of western New York will remember "11-11-11" as a beautiful fall day, somewhat windy, but with the mercury between 65 and 70 F. The day is all the more memorable because the Weather Bureau prophecied snow and only about 50 per cent. of the average temperature. It was still warm and pleasant at bed time and at two o'clock the next morning—as an emer-till nearly noon of the 12th, when the prophecy came true. The Christmas snow was similarly delayed for several days.

From the standpoint of the mariner and the farmer, the weather forecasts are reasonably reliable, occasional bad breaks being apparently unavoidable. But the health, comfort and pocket book of the average citizen require a much narrower margin of error and a more definite prophecy of the time at which weather will change. In the individual case, this requirement may seem trivial as compared with the value of crops and of ships, including their freight and passengers. A poor servant spoils ten dollars' worth of clothes in an unexpected rain quite local in its area. An overworked seamstress, overcautious, loses an infrequent chance for a summer day's outing because a storm is prophecied, which does not come off for a day or two. A business man with mild chronic nephritis goes to his office without an overcoat on a warm morning, is chilled through on his return, has an axacerbation of his renal condition, perhaps a pneumonia, and dies within a few days or has an expensive sickness and his life is shortened by two or three years. Some one else puts on heavy underclothing at the Weather Bureau's warning, swelters on a hot day, takes cold when the evening chills his body moistened with sweat and develops rheumatism. Multiply these trivial occurrences by, say, 50 million corresponding to the part of the population which is not directly interested in crops and maritime commerce and they are rather important factors in the incidence of disease, mortality and longevity, even the financial independence of the people.

We have no disposition to find fault with the Weather Bureau. Broadly speaking, for large areas and with a time limit of 24 hours—although we have seen the reports steadily prophecy bad weather for three or four days before it finally came—there has been developed a high degree of accuracy. But, for at least two-thirds of the population, what is needed is not a glittering generality for a large area, to be fulfilled in the course of a day or two, but a definite statement good for a half day or a few hours, for a few square miles and when this can not be made, a candid confession that a prophecy is impossible.

In our experience, local authorities, basing predictions on general observations and modifying them in accordance with their familiarity with peculiar local conditions, can almost invariably make a reliable forecast of the weather in accordance with the practical needs of the great mass of the people. They frequently know that the published reports are erroneous or, at least misleading, so far as immediate local conditions are concerned. The published reports come from headquarters and local stations are not allowed to publish their own forecasts.

With all due respect to the Weather Bureau, we venture to call attention to the fact that dependable prophecies, for local areas and single days or half days are a practical need of much greater importance than discipline or red tape.

